# Immediate Cementless Hemiarthroplasty for Severe Destructive Glenohumeral Tuberculous Arthritis

**DOI:** 10.1155/2013/426102

**Published:** 2013-09-19

**Authors:** Suriya Luenam, Arkaphat Kosiyatrakul

**Affiliations:** Department of Orthopaedics, Phramongkutklao Hospital and College of Medicine, 315 Ratchawithi Road, Bangkok 10400, Thailand

## Abstract

The glenohumeral joint tuberculosis (TB) is rare as compared with other joints. Plaster immobilization, arthrodesis, and resection arthroplasty have been proposed as the additional treatments with anti-TB medications in severe destructive arthritis. To our knowledge, however, the surgical treatment with shoulder arthroplasty has never been reported. We present two cases of active TB with unsalvageable glenohumeral joint. The cementless hemishoulder arthroplasties were performed immediately following the radical debridement. Anti-TB medications were given for 12 months after the surgery. Postoperatively, the patients were satisfied with the rapid symptomatic relief and significant functional recovery. With the follow-up period of 5 years, the operative results were still satisfactory and the reactivation of the infection was not detected.

## 1. Introduction

Tuberculosis (TB) continues to be a major health problem worldwide. The incidence of TB has increased since 1985, most likely due to the increase in HIV infection. Osteoarticular TB is an infrequent form of the disease, accounting for approximately 1–3% of all TB cases [1]. The incidence of osteoarticular TB affecting the shoulder is 1–2.8% of osteoarticular TB [2].

Owing to the insidious onset and nonspecific features, the diagnosis of articular TB is sometimes delayed until considerable cartilage and bone destruction occurs [3]. In an advanced stage of destruction, additional treatments were proposed in conjunction with anti-TB medications [3–12]. Immobilization of the shoulder until ankylosed in a functional position has been recommended to prevent or correct the adduction deformity [5–7]. Arthrodesis and resection arthroplasty are indicated when disabling deformity is persistent. Even though possibly providing the pain relief, these treatments commonly result in poor function and limited range of motion [8–12].

Based on our literature reviews, no report with regard to the treatment of active TB with primary shoulder arthroplasty has been described. We present two cases of active TB infection of the shoulder with advanced arthritis. Both were successfully managed by the immediate one-stage cementless hemiarthroplasty followed by anti-TB medications for 12 months.

## 2. Case Report

### 2.1. Case 1

A 50-year-old, right-handed, male chef presented in our clinic with a five-year history of right shoulder pain which was insidious in onset and gradually progressive. The range of shoulder movement was also increasingly restricted. He also had the decreased appetite and slow weight loss but no history of fever or other constitutional symptoms. There was no history of contact with tuberculosis or any predisposing conditions including rheumatoid arthritis, chronic steroid use, pulmonary diseases, and sexually transmitted disease. Pain and stiffness had increased in severity enough to restrict all day-to-day activities for the past one year.

Physical examination revealed that the patient was markedly wasted, slightly pale, afebrile, and anicteric. There was neither tense overlying skin nor discharging sinus in the shoulder area. The shoulder movements in the abduction, forward flexion, and external and internal rotation planes were marked restricted. Neither of these could go beyond 30 degrees from the rest position and mostly came from scapulothoracic motion. 

Laboratory examination revealed haemoglobin of 10 gm%, a total leucocyte count of 10,000 cm^3^ with 47% lymphocytes, 50% polymorphs, and 3% eosinophils. Erythrocyte sedimentation rate (ESR) was 33 mm/h. HIV screening test was seronegative. Chest X-ray was normal.

**Figure 1:**
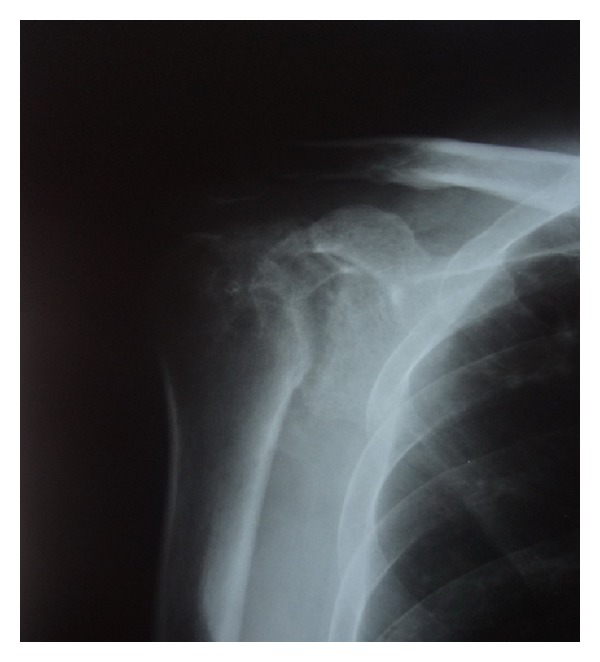
A plain radiograph showing severe glenohumeral joint destruction.

Plain radiographs showed the advanced arthritic change of the glenohumeral joint with juxta-articular osteopenia. Progressive collapse of a humeral head was apparent when compared with the previous X-rays (Figure 1). A computerized tomography (CT) scan demonstrated the severe arthritis with multiple cavity defects of the humeral head and glenoid. The diagnosis of TB infection was confirmed by histologic examination of the specimens obtained by CT-guided biopsy. The acid-fast bacilli with epithelioid histiocyte granulomas surrounded by lymphocytes and other mononuclear inflammatory cells were presented.

**Figure 2:**
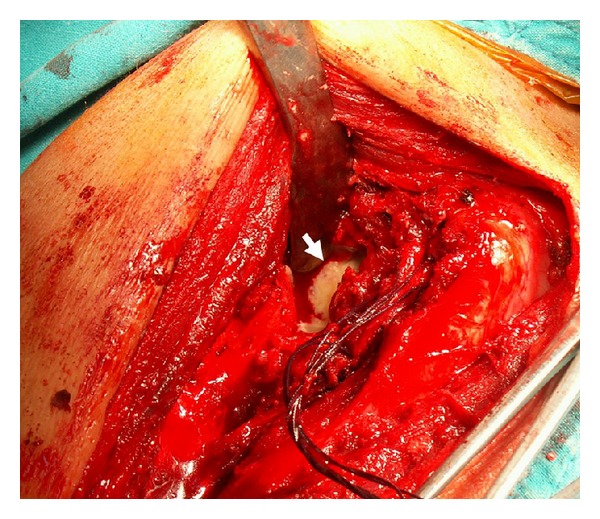
Intraoperative finding of the caseous material in front of the glenoid following the humeral head resection (white arrow).

**Figure 3:**
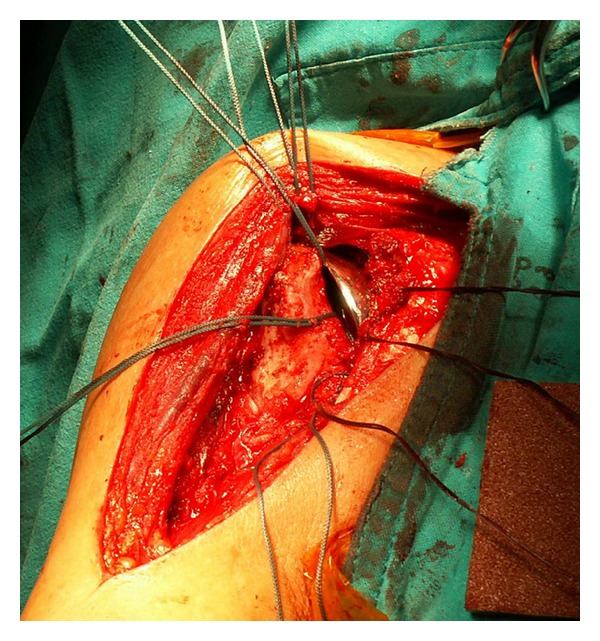
Immediate cementless stem humeral head arthroplasty was performed.

Intraoperatively, the deltopectoral approach was used to assess the glenohumeral joint. The extensive cartilage and bone destruction of the humeral head surrounded with the infective granulation tissues was observed. All rotator cuff tendons were intact but the supraspinatus tendon was very thin and fraying at the insertion. After the humeral head was resected, a large amount of the caseous material around the glenoid was found (Figure 2). The infected tissues as well as the unviable bones were completely removed. The humeral head arthroplasty was performed using a porous-coated cementless press-fit stem (Global Advantage Shoulder Arthroplasty System, DePuy Orthopaedics, Inc., Warsaw, IN, USA) (Figure 3). 

 The patient was given an anti-TB regimen with rifampicin (10 mg/kg), isoniazid (5 mg/kg), pyrazinamide (20 mg/kg) and ethambutol (15 mg/kg) for 14 days preoperatively and 2 months postoperatively. Rifampicin and isoniazide were continued until 12 months after surgery. Periodic and regular blood tests were done to check the potential toxicity of these drugs and the patients' visual acuity was also checked regularly.

**Figure 4:**
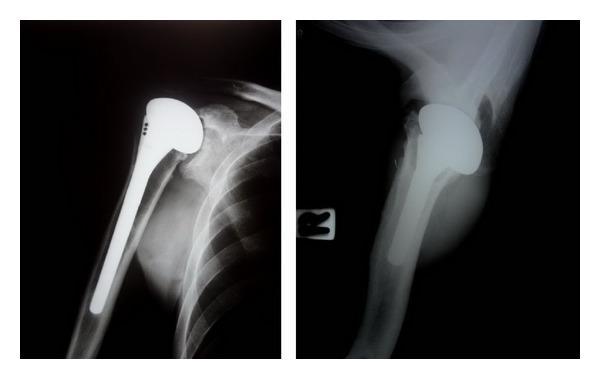
The follow-up radiographs at 60 months postoperatively.

The duration of postoperative followup was 60 months. The patient was satisfied with the surgical outcomes because of the rapid pain relief and significant improved physical function. ESR became normal, less than 15 mm/h in 4 months postoperatively. The constant scores of pain, ability of daily living, range of motion, and strength increased from 26 points at preoperative day to 70 points at 12-month followup. Pain symptom was also much improved as seen from the significant reduction of visual analogue scale (VAS) pain score from 8 to 1. The active shoulder abduction increased to 70 degrees. The shoulder forward flexion increased to 95 degrees. External and internal rotations increased to 50 degrees and to 55 degrees, respectively. At 60 months after surgery, the patient remains satisfied with his level of comfort and functional outcome. He had only minimal aching with heavy use of the upper extremity, but no other pain. The constant score was 72. The follow-up radiographs did not show any evidence of reactivation of the infection, component loosening, or osteolysis. The complete bone ingrowth of the humeral stem was visualized. However, the superior migration of the humeral head due to the rotator cuff insufficiency is noted (Figure 4). Gradual erosion of the glenoid conforming to the artificial humeral head was observed.

### 2.2. Case 2

An otherwise healthy 42-year-old, right-handed, male baker had the symptoms of gradually progressive pain and restricted movement in his right shoulder for 3 years. Pain had increased intensely for the past 6 months. The right shoulder presented mild swelling. The abduction, forward flexion, and external and internal rotation were restricted to 30 degrees which was mainly from the scapulothoracic motion. 

**Figure 5:**
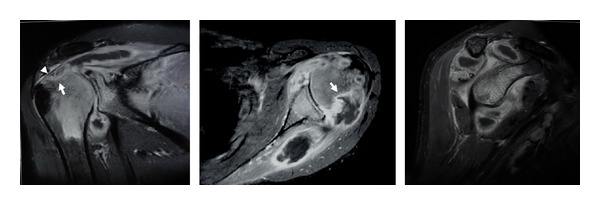
MRI demonstrating a large abscess surrounding the humeral head and extending into the supraspinatus and infraspinatus muscle substance. Significant bone erosion in the superior portion of the humeral head (white arrow) and thinning of the supraspinatus tendon (white arrow head) were revealed.

 The patient has a negative HIV screening test and normal chest X-ray. The complete blood count showed haemoglobin of 10 gm%, a total leucocyte count of 11,000 cm^3^ with 44% lymphocytes, 55% polymorphs, and 1% eosinophil. ESR was 40 mm/h. A small crescent osteolytic lesion just medial to the tip of greater tuberosity appeared on the plain shoulder X-rays. Magnetic resonance imaging (MRI) demonstrated generalized cartilage destruction with altered signal intensity of the proximal humerus. There was a large abscess surrounding the humeral head extending into the supraspinatus and infraspinatus muscle substance. Significant bone erosion in the superior portion of the humeral head and thinning of the supraspinatus tendon were revealed (Figure 5).

 He had the open biopsy done through the posterior aspect of the shoulder to confirm the diagnosis of tuberculosis before a referral to our department. The patient received four anti-TB drugs including rifampicin (10 mg/kg), isoniazid (5 mg/kg), pyrazinamide (20 mg/kg), and ethambutol (15 mg/kg) immediately after the surgery for 6 weeks. However, he reported no improvement of pain and the shoulder became even more swollen with local rise in temperature. The ESR was rising to 65 mm/h. Plain radiography showed a progressive humeral head destruction. A larger and increasing number of osteolytic lesions were obvious. 

 A thorough debridement of the abscess and infected tissue was achieved though the combined deltopectoral and posterior approaches. Severe articular erosion and bone loss of the humeral head were apparent. Following the head resection, complete removal of the extending abscess in the muscle substance around the glenoid was obtained. Significant damage of the rotator cuff muscles was noticeable especially the supraspinatus. The immediate cementless shoulder hemiarthroplasty was performed. The same postoperative anti-TB regimen as Case 1 was prescribed for 12 months. 

**Figure 6:**
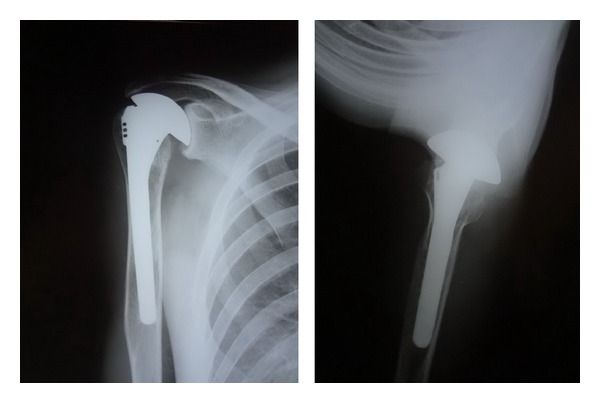
The follow-up radiographs at 60 months postoperatively.

 Postoperatively, pain symptom was rapidly decreased. The ESR was lower than 15 mm/h in 5 months. At 12-month followup, the constant scores increased from 26 points at preoperative day to 72 points. VAS pain score was reduced from 8 to 1. The active shoulder abduction increased to 75 degrees. The shoulder forward flexion increased to 95 degrees. External and internal rotations increased to 70 degrees and 55 degrees, respectively. At the 60 months after the operation, the patient was still satisfied with the result and able to tolerate strenuous work. The constant score was 74. There was no evidence of reactivation of TB or loosening of the implant. However, the narrowing of the acromiohumeral interval and medial glenoid erosion conforming to the metal humeral head was observed on follow-up X-rays (Figure 6). 

## 3. Discussion

 Glenohumeral joint TB is rare and delayed diagnosis is common. If the diagnosis is made after the disease has progressed beyond the stage of arthritis, the salvage treatments are including immobilization, arthrodesis, and resection arthroplasty [5–13]. To our knowledge, this is the first report of shoulder arthroplasty for the treatment of TB. 

 In shoulder joint TB with the presence of a severe cartilage and bone destruction, immobilization in a position of function until ankylosis occurs was recommended as a first line treatment by many authors [5–7]. Previous reports have demonstrated the immobilization could prevent the disabling stiffness in adduction; however, the affected shoulder generally healed with loss of movement. Martini et al. reported the treatment outcome of immobilization in 10 cases of shoulder joint tuberculosis [7]. External rotation was restricted to less than 20° in all shoulders and the abduction was less than 60° in 3 shoulders. On the contrary, Murphy and Wood advocated the shoulder arthrodesis in preference to the long period of immobilization. They believed the fibrous ankylosis from the immobilization method is generally insecure. From its own weight, the arm gradually loses its desirable abducted position and ultimately comes to rest in a poor functional position [12]. Although the arthrodesis usually provides a pain relief, the postoperative shoulder function is limited especially in activities that require arm rotation [8–10]. Shoulder resection arthroplasty can preserve the motion of glenohumeral joint. Unfortunately, this operation still leaves the patient with a severely restricted movement because the fulcrum of the shoulder is lost [10, 14, 15]. Many reports suggested that resection arthroplasty held no benefit over immobilization for shoulder joint TB [7, 9, 11]. However, in the largest case series of shoulder joint tuberculosis, 52% of patients required humeral head resection to control the infection [13].

Owing to the ability to restore the glenohumeral anatomy and motion, the arthroplasty is generally considered prior to proceeding with arthrodesis or resection arthroplasty in noninfective arthritis [8, 10]. Historically, the arthroplasty was not advocated in TB due to the concerns of continuing infection [13]. On the other hand, the current in vitro studies demonstrated that *Mycobacterium* rarely adheres to a metal surface and has little or no biofilm formation making it susceptible to anti-TB chemotherapy [16]. In addition, the recent evidence suggests that arthroplasty can be performed as long as adequate perioperative chemotherapy is maintained [6]. With respect to the hip and knee TB arthritis, immediate prosthetic arthroplasty for the treatment of advanced active tuberculous arthritis has been described in several case series (Table 1) [17–23]. The authors suggested that this is a safe procedure providing rapid symptomatic relief and functional improvement with low risk of reactivation. However, anti-TB medication and adequate surgical debridement are very important in ensuring success.

TB reactivation following the shoulder arthroplasty was previously described in two single case reports [24, 25]. Failure to control the infection and subsequent prosthetic removal is likely attributed to the initial missed diagnosis and lack of the anti-TB chemotherapy. However, the appropriate duration of anti-TB chemotherapy for primary prosthetic arthroplasty in active TB remains controversial. The Centers for Disease Control recommend 6 to 9 months for osteoarticular TB [26] whereas the protocols in many institutes recommended that treatment be continued for 12 to 18 months [19, 21, 22]. In the present report, both patients received the anti-TB medication for 12 months postoperatively. No reactivation of the disease occurred at 5-year followup. There is also no consensus regarding the starting time. Some authors believe that preoperative anti-TB medication may play an important role in reducing recurrence and recommend preoperative medication ranging from 1 week to 1 year before the surgery [19, 21–23, 27]. Further study with statistical analysis of its efficacy and optimal duration and starting time will be needed. 

As the infected tissue should be curetted out and debrided completely to eradicate the disease, a CT scan or MRI study may provide useful information on the extent of abscess spread [19–22]. In the present report, we also found that complete removal of the humeral head through the deltopectoral approach allowed full access to the abscess surrounding the glenoid. However, additional incision was required for thorough debridement of the intramuscular abscess of supraspinatus and infraspinatus. 

From the previous reports of active hip TB, the reactivation was not influenced by the presence of cemented or cementless implants [20, 21, 23]. However, cementless prostheses were more commonly used [19, 22]. We performed the cementless shoulder arthroplasty in our patients because the patients had adequate proximal humeral bone stock. In addition, if a patient has the reactivation, we believed the debridement and revision surgery is easier when using the cementless prosthesis as there is no retention of the cement mantle in the humeral canal. The bone ingrowth into the humeral stem was observed in both patients on the follow-up radiographs and the reactivation was not detected.

Due to the massive damage of the supraspinatus and infraspinatus, the only available prosthesis options are the hemiarthroplasty and reverse shoulder arthroplasty. The total shoulder arthroplasty is contraindicated because of the high risk of glenoid component loosening from the superior eccentric loading what is known as “rocking horse” phenomenon [28, 29]. The reverse shoulder prosthesis is a logical solution for the cuff deficient arthritic shoulder [29]. This prosthesis creates the fixed center of rotation and allows the deltoid to elevate arm despite the absence of functioning rotator cuff. However, the complication and revision rates are high. As the functional revision options are limited in case of failure, a careful patient selection is suggested. The Food and Drug Administration (FDA) recommends that reverse shoulder arthroplasty (RSA) be performed in patients 70 years or older. We decided to use the hemi-shoulder implants because both cases in our reports are younger active patients with expectations for heavy shoulder use. In the treatment of cuff deficient arthritic for other destructive shoulder disorders, the satisfactory outcomes of the hemishoulder arthroplasty have been published [28, 30–32]. In the present report, gradual conforming of the glenoid with the artificial humeral head proved that the motion occurs at the glenohumeral joint. The progression of glenoid wear and superior migration of the humeral head were noticed but did not worsen the clinical results of our patients in the 5-year followup. However, longer followup is necessary to determine the long-term results. 

Immediate cementless hemiarthroplasty seems to be a valid alternative for the treatment of glenohumeral joint tuberculosis with severe arthritis. The present report has shown the satisfactory outcome with no reactivation of TB infection. Partially restoring the fulcrum and preserving motion at the glenohumeral joint with the humeral head replacement may provide benefit for an increased shoulder movement, especially the rotation. While the scores for the pain relief and functional improvement have been excellent, the outcome measures for range of motion and strength were modest and inferior to those seen in primary osteoarthritis and rotator cuff deficiency [28, 32, 33]. A probable explanation might be that the extensive damaged rotator cuff and consequent adhesion from the long-standing infection impaired the ultimate results. 

## 4. Conclusion

Based on our patient outcomes, a primary single-stage cementless hemishoulder arthroplasty can be safely performed in active advanced osteoarticular TB of the shoulder for providing symptomatic relief and functional improvement. There is no need to wait to achieve the quiescent stage of the disease similar to several studies reported in TB hip and knee. 

## Figures and Tables

**Table 1:** Summary of the reports of immediate arthroplasty for active TB arthritis in the hip and knee.

Author(s)	Infected joint	Number of cases	Average follow-up period (months)	Results		Reactivation (cases)	Reference
(1) Gale and Harding	Knee	1	120	Satisfactory clinically and radiographically		None	[17]
(2) Wray and Roy	Knee	2	39	Satisfactory clinically and radiographically		None	[18]

				Harris Hip score			
				Preop	Postop		

(3) Yoon et al.	Hip	7	57.6	40	90	None	[19]
(4) Wang et al.	Hip	6	49	26.8	94.2	None	[20]
(5) Neogi et al.	Hip	12	41	38	88	None	[21]
(6) Oztürkmen et al.	Hip	9	67.2	37.3	94.8	None	[22]
(7) Kim et al.	Hip	4	33.3	57	86.3	None	[23]

## References

[1] Malaviya AN, Kotwal PP (2003). Arthritis associated with tuberculosis. *Best Practice and Research*.

[2] Tuli SM (2004). Tuberculosis of the shoulder. *Tuberculosis of the Skeletal System*.

[3] Sequeira W, Co H, Block JA (2000). Osteoarticular tuberculosis: current diagnosis and treatment. *American Journal of Therapeutics*.

[4] Tuli SM (2002). General principles of osteoarticular tuberculosis. *Clinical Orthopaedics and Related Research*.

[5] Sankaran B (1993). Tuberculosis of bones & joints. *Indian Journal of Tuberculosis*.

[6] Shrestha OP, Sitoula P, Hosalkar HS, Banskota KA, Spiegel DA (2010). Bone and joint tuberculosis. *University of Pennsylvania Orthopaedic Journal*.

[7] Martini M, Benkeddache Y, Medjani Y, Gottesman H (1986). Tuberculosis of the upper limb joints. *International Orthopaedics*.

[8] Clare DJ, Wirth MA, Groh GI, Rockwood CA (2001). Current concepts review: shoulder arthrodesis. *Journal of Bone and Joint Surgery A*.

[9] Kapukaya A, Subasi M, Bukte Y, Gur A, Tuzuner T, Kilnc N (2006). Tuberculosis of the shoulder joint. *Joint Bone Spine*.

[10] Cofield RH (1985). Shoulder arthrodesis and resection arthroplasty. *Instructional Course Lectures*.

[11] Mangwani J, Gupta AK, Yadav CS, Rao KS (2001). Unusual presentation of shoulder joint tuberculosis: a case report. *Journal of Orthopaedic Surgery*.

[12] Murphy JA, Wood C (1941). Tuberculosis of the shoulder: a report of four cases treated by operative fusion. *The Journal of Bone and Joint Surgery A*.

[13] Richter R, Nübling W, Schulz HJ, Köhler G (1986). Tuberculosis of the shoulder joint. *Zeitschrift für Orthopädie und Ihre Grenzgebiete*.

[14] Rispoli DM, Sperling JW, Athwal GS, Schleck CD, Cofield RH (2007). Pain relief and functional results after resection arthroplasty of the shoulder. *Journal of Bone and Joint Surgery B*.

[15] Braman JP, Sprague M, Bishop J, Lo IK, Lee EW, Flatow EL (2006). The outcome of resection shoulder arthroplasty for recalcitrant shoulder infections. *Journal of Shoulder and Elbow Surgery*.

[16] Ha K, Chung Y, Ryoo S (2005). Adherence and biofilm formation of *Staphylococcus epidermidis* and *Mycobacterium tuberculosis* on various spinal implants. *Spine*.

[17] Gale DW, Harding ML (1991). Total knee arthroplasty in the presence of active tuberculosis. *Journal of Bone and Joint Surgery B*.

[18] Wray CC, Roy S (1987). Arthoplasty in tuberculosis of the knee. Two cases of missed diagnosis. *Acta Orthopaedica Scandinavica*.

[19] Yoon TR, Sung MR, Setyagung BS, Sung TJ, Jong KS (2005). Immediate cementless total hip arthroplasty for the treatment of active tuberculosis. *Journal of Arthroplasty*.

[20] Wang Y, Wang J, Xu Z, Li Y, Wang H (2010). Total hip arthroplasty for active tuberculosis of the hip. *International Orthopaedics*.

[21] Neogi DS, Yadav CS, Kumar AK, Khan SA, Rastogi S (2010). Total hip arthroplasty in patients with active tuberculosis of the hip with advanced arthritis. *Clinical Orthopaedics and Related Research*.

[22] Oztürkmen Y, Karamehmetoğlu M, Leblebici C, Gökçe A, Caniklioğlu M (2010). Cementless total hip arthroplasty for the management of tuberculosis coxitis. *Archives of Orthopaedic and Trauma Surgery*.

[23] Kim Y-H, Han D-Y, Park B-M (1987). Total hip arthroplasty for tuberculous coxarthrosis. *Journal of Bone and Joint Surgery A*.

[24] Hattrup SJ, Bhagia UT (2008). Shoulder arthroplasty complicated by *Mycobacterium tuberculosis* infection: a case report. *Journal of Shoulder and Elbow Surgery*.

[25] Palacios EL, Baraia-Etxaburu J, Gutiérrez-Macías A, Teira R, Santamaría JM (2002). Infection of shoulder joint prosthesis by *Mycobacterium tuberculosis*. *Enfermedades Infecciosas y Microbiologia Clinica*.

[26] Blumberg HM, Burman WJ, Chaisson RE (2003). American thoracic society/centers for disease control and prevention/infectious diseases society of America: treatment of tuberculosis. *American Journal of Respiratory and Critical Care Medicine*.

[27] Jupiter JB, Karchmer AW, Lowell JD, Harris WH (1981). Total hip arthroplasty in the treatment of adult hips with current or quiescent sepsis. *Journal of Bone and Joint Surgery A*.

[28] Williams GR, Rockwood CA (1996). Hemiarthroplasty in rotator cuff-deficient shoulders. *Journal of Shoulder and Elbow Surgery*.

[29] Boileau P, Watkinson DJ, Hatzidakis AM, Balg F (2005). Grammont reverse prosthesis: design, rationale, and biomechanics. *Journal of Shoulder and Elbow Surgery*.

[30] Ramamohan N, Kelly IG (2002). Joint replacement in the rheumatoid shoulder. *Current Orthopaedics*.

[31] Park JH, Park JW, Shin JS, Lee JM, Lee JI (2012). Hemiarthroplasty in a patient with pigmented villonodular synovitis of the shoulder. *Orthopedics*.

[32] Sanchez-Sotelo J, Cofield RH, Rowland CM (2001). Shoulder hemiarthroplasty for glenohumeral arthritis associated with severe rotator cuff deficiency. *Journal of Bone and Joint Surgery A*.

[33] Rispoli DM, Sperling JW, Athwal GS, Schleck CD, Cofield RH (2006). Humeral head replacement for the treatment of osteoarthritis. *Journal of Bone and Joint Surgery A*.

